# The microbiome of the built environment and mental health

**DOI:** 10.1186/s40168-015-0127-0

**Published:** 2015-12-17

**Authors:** Andrew J. Hoisington, Lisa A. Brenner, Kerry A. Kinney, Teodor T. Postolache, Christopher A. Lowry

**Affiliations:** Department of Civil and Environmental Engineering, US Air Force Academy, 2354 Fairchild Dr. Suite 6H-161, Colorado Springs, CO 80840 USA; Rocky Mountain Mental Illness Research Education and Clinical Center (MIRECC), University of Colorado Anschutz Medical Campus, 1055 Clermont Street, Denver, CO 80220 USA; Civil, Architectural and Environmental Engineering, University of Texas Austin, 402 E. Dean Keeton Street, Austin, TX 78712-1085 USA; University of Maryland School of Medicine, Baltimore MD, Rocky Mountain MIRECC and VISN 5 MIRECC, 685 W. Baltimore Street, Baltimore, MD 21201 USA; Department of Integrative Physiology and Center for Neuroscience, University of Colorado Boulder, 1725 Pleasant Street, Boulder, CO 80309-0354 USA

**Keywords:** Bioinformed design, Built environment, Disease, Inflammation, Mental health, Microbiome, Neuropsychiatric disease, Psychiatric, Stress

## Abstract

The microbiome of the built environment (MoBE) is a relatively new area of study. While some knowledge has been gained regarding impacts of the MoBE on the human microbiome and disease vulnerability, there is little knowledge of the impacts of the MoBE on mental health. Depending on the specific microbial species involved, the transfer of microorganisms from the built environment to occupant’s cutaneous or mucosal membranes has the potential to increase or disrupt immunoregulation and/or exaggerate or suppress inflammation. Preclinical evidence highlighting the influence of the microbiota on systemic inflammation supports the assertion that microorganisms, including those originating from the built environment, have the potential to either increase or decrease the risk of inflammation-induced psychiatric conditions and their symptom severity. With advanced understanding of both the ecology of the built environment, and its influence on the human microbiome, it may be possible to develop bioinformed strategies for management of the built environment to promote mental health. Here we present a brief summary of microbiome research in both areas and highlight two interdependencies including the following: (1) effects of the MoBE on the human microbiome and (2) potential opportunities for manipulation of the MoBE in order to improve mental health. In addition, we propose future research directions including strategies for assessment of changes in the microbiome of common areas of built environments shared by multiple human occupants, and associated cohort-level changes in the mental health of those who spend time in the buildings. Overall, our understanding of the fields of both the MoBE and influence of host-associated microorganisms on mental health are advancing at a rapid pace and, if linked, could offer considerable benefit to health and wellness.

## Background

The influence of the built environment on the mental health of building occupants has been documented including relationships between housing quality, occupant density, noise, indoor air quality, and mental health [1]. One major relationship not yet elucidated is the connection between indoor microorganisms and the mental health of human occupants. Recent advances in DNA sequencing technologies and associated cost savings have led to an expansion of research on microorganisms observed indoors, collectively called the microbiome of the built environment (MoBE). Led in part by the contributions and organization of the Alfred P. Sloan Foundation, building scientists and microbial ecologists are collaborating to (1) investigate the influence of architecture on the MoBE [2, 3], (2) establish a community of cross-disciplinary researchers [4], and (3) develop tools required for data analysis and visualization [5, 6]. To our knowledge, the MoBE has yet to be evaluated with respect to mental health outcomes.

Preclinical evidence strongly supports the important influence of the human microbiome (e.g., microorganisms localized to the gut, skin, and other organs) on systemic inflammation [7–11], autoimmunity [12], blood-brain barrier function [13], neuroinflammation [14], cognitive function [15, 16], and emotional behavior [16–19]. Specifically, there is an *increasing appreciation* regarding the potential association between inflammation and mental health, ranging from wellness to neuropsychiatric disease [20–24]. This association is of concern as evidence suggests that chronic inflammatory disorders are increasing in high-income countries. One factor contributing to this increase is thought to be failing immunoregulation, attributable to reduced exposure to the microbial environment within which the mammalian immune system co-evolved [23]. We, along with others, have proposed that faulty immunoregulation is also driving increases in some psychiatric disorders [23, 25–27]. The full development of secondary lymphoid tissues and a diverse lymphocyte repertoire after birth requires signals from microbial components [28], while further microbial signals later in life drive balanced expansion of effector T cell populations [29] and regulatory T cells [30, 31]. The organisms most responsible for these effects are those with which mammals co-evolved, including the following: (1) the commensal/symbiotic microbiota [30, 31]; (2) certain “old infections,” including *Helicobacter* species, that were present throughout life in evolving human hunter-gatherer populations [25, 32]; and (3) organisms from the natural environment with which humans were inevitably in daily contact throughout evolution [33–35]. Immunoregulation is compromised in modern high-income settings in part because contact with these three categories of immunoregulatory organisms is reduced [25].

Several society-level shifts, such as those noted below, have and will continue to lead to an increasing disconnect between humans, the outdoor environment, and the microorganisms with which humans co-evolved [36–38]. In the USA, individuals typically spend nearly 90 % of their time in the built environment [39]. Comforts, such as air conditioning, have made geographic locations more hospitable for year-round living in the indoor environment and will likely drive increased time spent in the built environment [40]. Residential homes built in set parcels for subdivisions have an architecture that is typically not conducive to natural heating and cooling. To reduce energy costs, design and subsidized weatherization programs can further reduce outdoor exposures by making the homes less leaky and lowering air exchange rates [41]. This issue may be relevant to developing, as well as developed, countries. Migration from rural to urban environments in developing countries is occurring at a rapid pace [42], and it could lead to a reduction in exposure to environmental microorganisms, as already seen in industrialized nations.

**Fig. 1:**
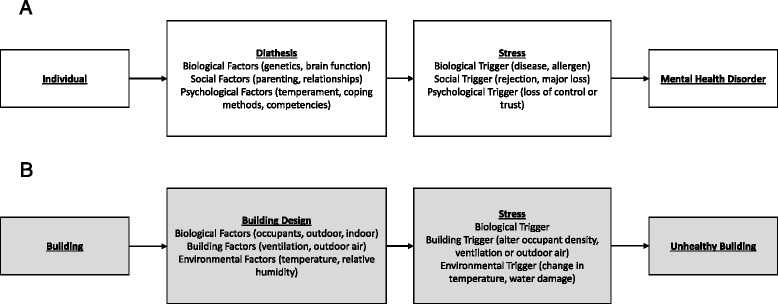
Parallels between individual diathesis-stress model (**a**) and potential unhealthy building model (**b**). Note that this comparison is not all inclusive of factors or triggers in either model

One popular model for conceptualizing the onset of psychiatric disorders is the stress-diathesis model [43, 44]. The model suggests that individuals have biopsychosocial vulnerabilities for developing mental health illnesses (diathesis) that can be realized through stressors. We contend that model has parallels to a model for unhealthy buildings (Fig. 1). That is, an unhealthy building can have design or operational flaws (diathesis) that under specific circumstances (stressors) create a predisposition to poor indoor air quality for the occupants. For example, sick building syndrome is a term used to describe an unhealthy building in which occupants living and working in that space are found to be suffering from acute negative outcomes which are not linked to a specific cause. This article expands upon how the MoBE and mental health fields can and should be integrated. Specifically, we will investigate (1) the effects of the MoBE on the human microbiome and (2) potential opportunities for manipulation of the MoBE in order to improve mental health. In addition, we will propose future research directions, including strategies for assessment of changes in the microbiome of common areas of built environments shared by multiple human occupants and associated cohort-level changes in the mental health of those who spend time in the building. The purpose of this review is to initiate discussions that build new research efforts between building scientists, microbial ecologists, and clinical research psychologists and psychiatrists.

## Review

Microbial exposures early in life can have long-lasting impacts on the immune system including reducing inflammatory responses in adulthood [45]. One potential connection between the MoBE and mental health could be the influences of the MoBE on the human microbiome. For that influence to occur, it must be shown that microorganisms in the built environment are transferred to occupants. Researchers have also determined that reciprocal interactions occur between the host immune system and host microbiome. In a murine model, altered immune status, either induced by antagonism of complement component 5a receptor 1 (C5aR) or as observed in immunocompromised mice, was associated with decreases in host-associated skin microbial diversity and altered microbial community structure [46, 47]. Activation of the complement system typically functions as a defense mechanism against invading microorganisms, in part because C5a “primes” phagocytic cells and optimizes innate immune functional responses. Thus, as expected, altering the host immune response to microorganisms alters the host-associated microbial diversity and community structure. With the expansion of the biological field beyond traditional culturing, it is now clear that microbial residue can persist in the environment past a viable cell life [48, 49]. Indeed, microorganisms or microbial antigens, in addition to live, intact microorganisms, are sufficient to have important impacts on systemic immune function and inflammatory disease. For example, heat-killed preparations of mycobacteria have been shown to confer protection in allergy models [50]. In other words, there are reciprocal relationships between the host-associated microbiota and host immune function, and microorganisms do not have to be living, or even intact, in order to have important influences on health outcomes. This may be of interest to MoBE researchers who, to date, have used DNA sequencing that does not distinguish between live, dead but intact, or dead and structurally compromised microorganisms. However, methods using compounds like propidium monoazide (PMA) have shown promise for making these distinctions in qPCR studies [51–53]. Additionally, Kelley and Gilbert [48] suggested mRNA as a sequencing target to distinguish between live and dead microorganisms because RNA is rapidly degraded in the environment.

Current research has focused on how occupants alter the MoBE and have documented the transmission of microorganisms from human occupants to the air and surfaces within buildings [2, 3, 54–59]. Human skin microorganisms are likely a major source of indoor human-related microorganisms and have been observed in the MoBE in classrooms [60], households [57], and athletic environments [55]. The human occupants deposit skin microorganisms at a rate of 10^6^ airborne microbial cells per hour [61], but these microorganisms can decay and are replaced at a rapid rate on surfaces commonly in contact with humans [56]. Whereas the cutaneous membrane is completely exposed to the environment [62], mucosal surfaces of the bronchopulmonary system, gastrointestinal system, and genitourinary system each have their own microbiota, with potential for contributions from exposure to microorganisms from the MoBE [63–65]. The skin, mucosal surfaces, and the immune system are in constant communication to promote homeostasis in the human microbiome [11, 66, 67]. Research is sparse on whether these microbial communities, transferred from occupants to the air and surfaces within a building, are conveyed to other occupants. We think it is important to identify whether the existing MoBE can alter the occupants’ microbiome and, subsequently, mental health.

Studies that document transmission of microorganisms from reservoirs within the built environment to human occupants have historically focused on pathogens. For instance, nontuberculous mycobacteria (NTM) isolates recovered from water systems and showers have been matched to clinical isolates of NTM [68, 69]. Similarly, room humidifiers, whirlpools, air conditioning systems, and other sources have been identified as the indoor microbial reservoirs responsible for *Legionella* infections of human occupants [70–72]. While some indoor sources such as humidifiers provide a direct route of microbial transfer to humans [73, 74], other transfers can be more complex. For instance, in some cases, microorganisms such as *Staphylococcus aureus* originate from a human source but appear to spend time in an indoor reservoir before being transmitted back to other human occupants [75]. Thus, microorganisms can be transferred to and from occupants and environmental reservoirs within buildings but the level of bidirectional transmission for many microorganisms and microbial communities remains unknown. Of course, transmission of microorganisms from one human occupant to the next is also possible [76, 77], and this further complicates the analysis.

A practical consequence of improved understanding of relationships among the MoBE, host-associated microbiota, and mental health could be the development of a bioinformed design in the built environment (see commentary by Green [78]) to prevent negative mental health-related outcomes. This could be analogous to how access to green spaces can improve mental health outcomes [33]. Moreover, studies in rodents suggest that bioimmunomodulatory probiotics (e.g., *Lactobacillus reuteri*) have stress-protective effects and can reduce negative outcomes of gastrointestinal infections [16]. Preliminary results in mice suggest these probiotics can reduce anxiety and fear following oral or intravenous administration. Building materials or the built environment could be designed to facilitate exposure of human occupants to these types of beneficial probiotics. Probiotics in these instances might extend beyond a few microorganisms and instead include diversity in microbial communities. Microbial diversity has been linked to positive health outcomes, even if the causative agents are not yet delineated [79].

Research on microbial growth on different building materials is now being considered beyond moisture-damaged materials [80–82], but more studies are required to fully evaluate the range of construction practices and building types. The development of such a database of materials that support growth of beneficial microorganisms, in addition to those that support growth of potentially harmful microorganisms, would be of value to the community of researchers. However, to develop that database, knowledge of which microorganisms are beneficial is required. In the context of this review on mental health, we provide a summary in Table 1 of microorganisms that have been linked to positive mental health outcomes. In addition to materials, selection of microorganisms for bio-mediated construction procedures is a relatively new field in the last 10 years and could provide benefits for a bioinformed built environment.

**Table 1:** Beneficial microorganisms in mental health studies found in the built environment

Phylum/microorganism	Model	Environmental sources	Presence in MoBE	Mental health relevant findings^a^
*Actinobacteria*				
*Mycobacterium vaccae*	Human	Environmental saprophyte (soil, mud, water, grasses, decaying organic matter) [119–125]	Soil floors [123], reservoirs [125], well water [122, 126], cooling towers [126, 127], water distribution systems [128], household tap water [126, 129], moisture-damaged building materials [130], terraria [131], sewage [122], drainage pools [132], wastewater treatment plants [133]	Increased cognitive function, decreased pain in patients with advanced non-small-cell lung cancer [134]
	Mouse			Activation of brain serotonergic systems and antidepressant-like behavioral effects [135]; decreased anxiety/increased cognitive function [136]
*Bifidobacterium breve*	Mouse	Human commensal	Human and animal wastewater, wastewater treatment plants [137]	Increased cognitive function [138]; decreased anxiety-related behaviors [139]
*Bifidobacterium infantis*	Rat	Human commensal	Human and animal wastewater, wastewater treatment plants [137]	Reversal of depressive-like behavior following maternal separation [140]
*Bifidobacterium longum*	Human	Human commensal	Human wastewater, wastewater treatment plants [137]	Decreased anxiety and depressive symptoms in healthy volunteers (administered with *L. helveticus*) [141, 142]
	Mouse			Decreased-colitis associated anxiety [143, 144]; increased cognitive function [138]; decreased stress, anxiety- and depression-related behaviors [139]
*Bacteroidetes*				
*Bacteroides fragilis*	Mouse	Human commensal	Human and animal wastewater, wastewater treatment plants [145]	Developmental protection from some of the behavioral symptoms associated with autism spectrum disorder [146]
*Firmicutes*				
*Clostridium butyricum*	Human	Endospore-forming soil bacterium		Anxiolytic effects [147]
*Enterococcus faecium*	Mouse	Human commensal, wetlands [148]	Well water, human and animal wastewater, wastewater treatment plants [148]	Increased brain antioxidant markers [149]
*Lactobacillus casei*	Human	Human commensal, fermented foods [150]	Human and animal wastewater, wastewater treatment plants [150], office space (*Lactobacillus* spp.), bathroom surfaces (*Lactobacillaceae*) [151]	Improvement in anxiety symptoms in patients with chronic fatigue syndrome [152]; improved mood [153]
*Lactobacillus fermentum*	Rat	Human commensal, raw vegetables [154], fermented foods [150, 155]	Sewage [155], office space (*Lactobacillus* spp.) [156], bathroom surfaces (*Lactobacillaceae*) [151]	Decreased anxiety and inhibition of antibiotic-induced cognitive impairment [157]
*Lactobacillus helveticus*	Human	Fermented foods [150]	Office space (*Lactobacillus* spp.) [156], bathroom surfaces (*Lactobacillaceae*) [151]	Decreased anxiety and depressive symptoms in healthy volunteers (administered with *B. longum*) [141, 142]
	Rat			Improved cognitive function, decreased anxiety-related behavior [158]; prevention of stress-induced cognitive impairment and anxiety- and depressive-like responses [159]
	Mouse			Decreased anxiety-related behavior [160]; improved cognitive function, decreased anxiety-related behavior (administered with *L. rhamnosus*) [15, 161]
*Lactobacillus pentosus*		Fermented foods [150]	Sewage [155], office space (*Lactobacillus* spp.) [156], bathroom surfaces (*Lactobacillaceae*) [151]	Improved cognitive function [162]
*Lactobacillus reuteri*	Human	Human commensal, fermented foods [150]	Office space (*Lactobacillus* spp.) [156], bathroom surfaces (*Lactobacillaceae*) [151]	Increased workplace healthiness [163]
*Lactobacillus rhamnosus*	Mouse	Human commensal, fermented foods [150]	Sewage [155], office space (*Lactobacillus* spp.) [156], bathroom surfaces (*Lactobacillaceae*) [151]	Vagus nerve-dependent alterations in GABA receptor mRNA expression in brain, reduced anxiety- and depression-related behavior [17]; improved cognitive function, decreased anxiety-related behavior (administered with *L. helveticus*) [161, 164]
Probiotic cocktails				
*B. bifidum*, *B. lactis*, *L. acidophilus*, *L. brevis*, *L. casei*, *L. salivarius*, *L. lactis*	Human			Reduced cognitive reactivity to sad mood [165]
*B. animalis* subsp. *Lactis*, *Streptococcus thermophilus*, *L. bulgaricus*, *L. Lactis* subsp. *Lactis*	Human			Altered task-related response of brain networks involving affective, viscerosensory, and somatosensory cortices [166]
*L. acidophilus*, *B. lactis*	Human			Improved scores on anxiety, depression, and stress scales [167]
*L. casei*, *L. acidophilus*, *L. rhamnosus*, *L. bulgaricus*, *B. breve*, *B. longum*, *S. thermophilus*	Human			Improved scores on anxiety, depression, and stress scales [167]
VSL#3: *S. salivarius* subsp. *thermophilus*, *B. breve*, *B. infantis*, *B. longum*, *L. acidophilus*, *L. planarum*, *L. casei*, *L. delbrueckii* subsp*. bulgaricus*	Mouse			Decreased sickness behavior, decreased microglial activation [168]
*L. plantarum*, *L. curvatus*	Rat			Improved cognitive function [169]
*L. acidophilus*, *B. lactis*, *L. fermentum*	Rat			Improved cognitive function [170]
*L. helveticus*, *B. longum*	Rat			Decreased depressive-like behavior [171]

^a^Although mental health benefits of microorganisms are typcially strain dependent, we have not included strain information in order to simplify the table

However, as noted by Green, research to date is not yet sufficient to define interactions between microorganisms and the built environment, and the effects that manipulation of the MoBE could have on the occupants. Indeed, only recently have we begun to realize that indiscriminant sterilization of the built environment is not beneficial to occupants [83–85]. The science of either seeding the indoor environment with beneficial microorganisms or providing conditions that promote selective microbial growth is likely a distant reality in practice, and considerable research is required before researchers can recommend such practices. Any modifications would need to consider factors such as geographic location, seasons, building characteristics, occupant ages, health status, and behavior, and likely many other factors yet to be determined. Future work in this direction could provide considerable benefit in terms of mental health wellness. However, as noted by Logan [86], additional adverse environmental factors in urban environments, coined the gray space, could reduce the mental health benefits of changing the MoBE, especially in relation to those individuals who are socioeconomically disadvantaged. An example of an understudied topic is the interaction between the MoBE and the host-associated microbiomes among individuals living or working in close proximity. An initial study of a family observed homogenization of the gut microbiome across family members, at least in comparison to non-family members [87].

Given the importance of inpatient care on mental health, one specific built environment of interest in the relationship between microbiomes of the built environment is that of the hospital. Recently, a collaboration led by the University of Chicago has systematically explored the microbiome in a Chicago hospital from conception through the first year of operation [83]. The results of that work are not yet published, but other articles have identified methods to control microbial spread in a hospital environment including ventilation strategies [88, 89], cleaning techniques [90–92], and use of UV lamps [93–96]. Interestingly, since we do not have a clear understanding of the role of the built environment microbiome on mental health, it is impossible to state if those listed control measures and others are beneficial or harmful to the occupant. Additional research on the influence of the gut and non-gut microbiomes and mental health is required to provide better designs in hospital and treatment centers.

With a better understanding of the interactions between the MoBE and the host-associated microbiota of human occupants, research could proceed to identification of environmental microorganisms that are either beneficial or harmful to the mental health of individuals, thereby mitigating potential diatheses or stressors. Human microbial communities differ across the body, but identification of an individual by their microbial fingerprint can still be achieved through multi-kingdom metagenomics sequencing with an accuracy of over 80 % [62]. Therefore, if certain MoBE markers are determined to contribute to mental health conditions, researchers could use sampling of the built environment to detect potential negative mental health conditions of individuals in that environment. Identification of associations between occupants and the microbiome of their surroundings or personal items, referred to as microbiome fingerprinting, may be possible in the future. Lax et al. [97] utilized a supervised learning algorithm to successfully predict if a 16S rRNA sample was from a phone or a shoe. They sampled at three different geographically separated conferences and were able to predict which conference the samples were from. Another study of phones sampled the microbiome of both the phones and fingerprints and observed that 82 % of the dominant bacterial sequences was shared between a user and their phone [98]. Using the human microbiome project dataset, by averaging microbiomes over all 18 body sites, and by developing a metagenomic code, Franzosa et al. [99] determined that approximately 30 % of sequences were matched between two sampling events 30 to 300 days apart. The most stable microbiome was the gut, which likely does not significantly contribute to the MoBE outside of restrooms. As noted by that study, research on how the individual microbiomes differ across anatomical sites and longitudinally over time will be crucial for future microbiome fingerprinting efforts.

One important under-recognized contributory role to the MoBE is that of pets. Their microbiome is known to interact with that of their owners.  Exposure to pets during childhood has been associated with lower prevalence of allergic disease, potentially through increased pet-driven indoor exposure to saprophytic soil organisms with immunomodulatory potential (see Table 1). However, pets also carry indoors microorganisms with potential long-term detrimental effects on mental health. For example, Toxoplasma gondii (T. gondii) is a highly prevalent neurotropic parasite establishing latency in all warm-blooded animals including humans. In immunosuppressed patients and during pregnancy its effects can be devastating. In immunocompetent hosts “latent” T. gondii infection has been associated with mental illness [100] and suicide [101]. Even in individuals with no evidence of mental illness, T. gondii seropositivity has been associated with gender-specific trait impulsivity and aggression [102]. Infection with T. gondii occurs via ingestion of tissue bradyzoites from inadequately cooked/ processed meat, or via transmition of oocysts through exposure to cat litter or contaminated soil [103]. Cats, the permanent hosts of T. gondii, are implicated in the direct fecal transmission of the microorganism; pregnant women are advised to avoid exposure to domestic cat litter. Moreover, dogs carry oocysts indoors on their fur by seeking-out and rolling in cat feces and contaminated soil [104].

An extension of microbiome fingerprinting could involve examining the influence of stress on the human microbiome and MoBE. Individuals suffering from psychiatric disorders may have physiological differences that affect the skin [105] and mucosal [106, 107] microbiomes, which in turn may alter the MoBE. For example, Biagi et al. [108] found that 9 % of the total variability seen in the gut microbiome was related to proinflammatory cytokines IL-6 and IL-8, with this effect being predominately driven by *Proteobacteria*. As noted, a feedback loop between occupants, the built environment, and other occupants has been shown in nosocomial infections. That same feedback loop is likely occurring for non-pathogens although not as well studied to date. Nosocomial pathogens like *Staphylococcus aureus*, *Mycobacterium tuberculosis*, and *Clostridium difficile* can remain viable on dry surfaces for months [109, 110], although as already noted, microorganisms do not have to be viable to contribute to health outcomes.

## Conclusions

Despite the massive effort in the human microbiome project, researchers observed that variation in the healthy human microbiome was not well correlated with biometrics (gender, body temperature, blood pressure, etc.) and concluded that other factors might be important [111]. One such factor could be the MoBE that surrounds individuals. It is likely that the MoBE alters immune system function through influences on the host-associated microbiota and, therefore, could have an effect on the mental health of the occupants as presented in Fig. 1. Study designs developed by mental health professionals, building scientists, and microbial ecologists should begin to critically evaluate that idea. Furthermore, given the recent interest in the microbiome-gut-brain axis [112–115], if the MoBE does alters the gut microbiota, considerable research opportunities could follow for future studies of the relationships among the microbiota of the built environment, the host-associated microbiota, and mental health with the aim of intervening to decrease negative health-related outcomes. It is possible that intentional modification of the built environment to increase microbial biodiversity, or to increase exposure to immunoregulatory antigens or probiotics, would result in improved mental health conditions. Moreover, mental health and MoBE studies logically extend to autism spectrum disorders and other neurodevelopmental disorders, such as schizophrenia, that have been investigated with a human microbiome perspective [116–118].

There is credible evidence that these two fields, if linked, could improve future mental health outcomes for both the community at large (e.g., prevention), as well as those with psychiatric disorders (intervention). Research studies will provide the most benefit if discussions among investigators with multiple specialties are initiated in the study design phase. With a combined effort between the MoBE consortia and mental health professionals, a greater understanding of the relationship between the MoBE, the microbiome of human occupants, and mental health can occur at a more rapid pace.
